# The effect of ego-depletion on college students’ deceptive behavior: the role of anonymity and moral emotions

**DOI:** 10.3389/fpsyg.2025.1506966

**Published:** 2025-04-25

**Authors:** Lingxue Ren, Meng Ning, Zhihao Chen, Shaobo Zhang

**Affiliations:** ^1^School of Education Administration, Shaanxi Fashion Engineering University, Xi’an, China; ^2^School of Education Administration, Qiqihar University, Qiqihar, China; ^3^School of Government, East China University of Political Science and Law, Shanghai, China

**Keywords:** ego depletion, deceptive behaviors, anonymity, moral emotions, dual-systems model, self-control two-stage model, ego depletion process model

## Abstract

**Objective:**

The present study aimed to investigate the effects of anonymity and moral emotions on college students’ deceptive behavior under different ego depletion conditions.

**Methods:**

In Experiment 1, 120 college students were recruited and assigned to a 2 (ego depletion: high vs. low) × 2 (anonymity: anonymous vs. non-anonymous) between-participants design to examine the impact of anonymity on deceptive behavior under varying levels of ego depletion. In Experiment 2, 150 college students were recruited and assigned to a 2 (ego depletion: high vs. low) × 3 (moral emotions: positive vs. negative vs. neutral) between-participants design to investigate the effects of moral emotions on deceptive behavior under different ego depletion conditions.

**Results:**

In Experiment 1, results revealed a significant main effect of ego depletion: the high ego depletion group exhibited more deceptive behavior than the low ego depletion group, and their decision-making reaction times were shorter. The main effect of anonymity was also significant, with the anonymous group showing more deceptive behavior than the non-anonymous group. Moreover, a significant interaction effect was found; under high ego depletion conditions, the anonymous group exhibited greater deceptive behavior than the non-anonymous group (all *p* < 0.001). In Experiment 2, the main effect of moral emotions was significant: the positive moral emotion group exhibited less deceptive behavior than the negative moral emotion group, which in turn exhibited less deceptive behavior than the neutral emotion group. Additionally, a significant interaction effect was found, under high ego depletion conditions, the positive moral emotion group demonstrated less deceptive behavior than the negative moral emotion group, which, in turn, demonstrated less deceptive behavior than the neutral emotion group (all *p* < 0.001).

**Conclusion:**

The findings indicate that under high ego depletion conditions, college students engage in more deceptive behavior. Anonymity exacerbates the after-effects of ego depletion, leading to increased deception, whereas moral emotions help mitigate these after-effects and reduce deceptive behavior.

## Highlights

We found that ego depletion contributes to increased deceptive behavior among college students.
Anonymity exacerbates the aftereffects of ego depletion, further increasing deceptive behavior.
Moral emotions can mitigate the aftereffects of ego depletion and reduce deceptive behavior.

## 1 Introduction

Deceptive behaviors are pervasive in daily life, manifesting in various forms such as disguise, concealment, and fake actions (Lewis, 2015). Research in evolutionary psychology suggests that deceptive behaviors can enable individuals to gain wealth, fame, and opportunities within social groups (Jung and Lee, 2009). Although some forms of deception may be pro-social or aimed at enhancing interpersonal communication, the majority of deceptive behaviors are driven by self-interest and exhibit anti-social characteristics (DePaulo et al., 2003). Poter and Campbell (1999) defined deception as an act in which the deceiver leads the deceived to accept fabricated facts, ultimately achieving personal goals at the expense of the deceived. This study adopts this definition, positing that deception in real life is not merely a response to an error but a complex process involving attention, cognition, reasoned decision-making, and social interactions. Understanding the mechanisms underlying deceptive behaviors holds both practical and academic significance.

Despite the findings of prior studies indicating a potential link between self-depletion and deceptive behavior (Kouchaki et al., 2014), the underlying mechanisms remain to be fully elucidated. In situations where individuals find themselves in a state of ego-depletion, what strategies can they employ to mitigate the likelihood of deceptive behavior? Some studies have found that the environment in which an individual is located can affect the probability of their deceptive behavior (Nordgren and McDonnell, 2011). Some studies also have found that moral emotions have an impact on an individual’s deceptive behavior. Gilligan (1977), for instance, posits that evoking feelings of empathy and compassion in an individual can influence their moral judgments and social conduct. However, Nunner-Winkler and Sodian (1988) also found that 4–5 year old children still choose to shove others and take over their swings even though they know their behavior is against the rules and immoral. In recent years, researchers have focused intently on the influence of moral emotions on individual behavior, nevertheless, the findings of research in this domain have been inconclusive (Malti et al., 2009; Zhou et al., 2017), which is also the focus of this study.

### 1.1 Conceptual framework

**Figure 1:**
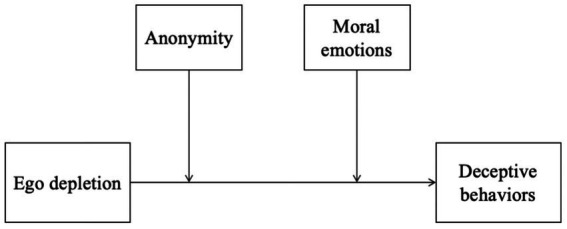
The theoretical framework of the variables.

In this study, we have developed a theoretical framework (shown in Figure 1) to examine the impact of anonymity and moral emotions on deceptive behaviors. The framework integrates insights from prior psychological research, which will be discussed in more detail below.

### 1.2 Ego depletion and deceptive behaviors

Self-control refers to the capacity of individuals to consciously regulate their impulses, desires, and behaviors (Baumeister et al., 1998). When immediate temptations conflict with long-term goals, individuals are able to suppress impulsive tendencies and align their actions with long-term needs (Baumeister et al., 1998). However, self-control is not always successful in real life. Failures in self-regulation, such as obesity and drug addiction, are common and can have detrimental effects on individual and societal well-being. The concept of ego depletion has long been explored in the self-control literature. In earlier studies, ego depletion is sometimes viewed as a process in which mental energy is consumed during self-control efforts (Baumeister et al., 1998), and at other times, it is defined as a state in which executive functions are impaired due to the depletion of psychological resources (Hagger et al., 2010). In this study, we employ the first definition, which believes that ego depletion is the process of gradual depletion of psychological energy in the period of self-activity, and the state in which the individual’s executive ability is reduced due to the depletion of psychological energy is called the aftereffect of ego depletion.

The influence of ego depletion on an individual’s deceptive behaviors can be explained in the dual-systems model of self-control proposed by Hofmann et al. (2009). According to the dual-systems model, the individual mainly relies on two systems to carry out a series of self-control, one is the subliminal-inspired dynamic system, also known as the hot system; the other is the consciously controlled deep-thought system, also known as the cold system, and the final behavior depends mainly on which of the two systems is currently more aroused. The hot system is activated intuitively, relying primarily on automatic, unconscious processing that does not demand cognitive resources, resulting in relatively shorter reaction times. In contrast, the cold system engages when an individual consciously identifies conflicts, monitors information, and inhibits behavior, its activation consumes self-control resources, leading to comparatively longer reaction times. When an individual makes a moral decision, the participation of the self-control system is required, and the cold system is dominant at this time, but ego depletion depletes the psychological energy of the individuals, failing self-control and then the hot system is dominant at this time, which leads to unethical behaviors like deception (Hofmann et al., 2012). In a study by Kouchaki et al. (2014), it was shown that people are more likely to engage in deceptive behaviors in the afternoon, because a morning of work and study depletes a large amount of psychological energy, which weakens people’s ability to restrain non-moral consciousness, and thus produces deceptive behaviors. Based on Fan et al. (2019), the present study categorizes deceptive behavior into two components: the number of deceptions and the tendency to deceive. In line with this approach, we propose Hypothesis that:

*H1: the higher the level of ego depletion, the greater the number of deceptions and the more severe the deception tendency, and the shorter the deception reaction time as well as the reaction time for reporting deception tendency (in ms).*

### 1.3 Ego depletion and deceptive behaviors: anonymity

The widespread implementation of street lighting in 19th-century urban landscapes can be attributed, in part, to its role in reducing the anonymity of nighttime environments. Bouman (1987) found that the anonymity conferred by darkness significantly influences individuals’ engagement in unethical behavior. Additionally, research suggests that the reduction of individuals’ unethical behavior in group settings primarily stems from individuals’ aversion to negative evaluations and potential losses imposed by others (Koch and Normann, 2005). However, in anonymous contexts, individuals’ unethical actions are less likely to be detected by others, thereby reducing the perceived risk of social and material consequences (Dillenberger and Sadowski, 2012). In the experimental study by Zhong et al. (2010a, 2010b), higher self-reported scores were associated with greater monetary rewards. The results revealed that participants’ self-reported scores in the dark room were significantly higher than those in a normal setting, yet there were no differences in their actual scores across the two conditions. This finding indicates that participants in the dark room engaged in deceptive behavior. Joinson (2003) found that the anonymity, invisibility, and psychological detachment afforded by online environments lower individuals’ self-regulation thresholds, thereby increasing the likelihood of engaging in socially deviant behaviors that deviate from normative constraints. Research by Piazza and Bering (2008) also suggests that anonymity diminishes feelings of shame, lowers self-control, and leads to more selfish and antisocial behaviors. So how does anonymity influence an individual’s level of self-control?

Based on the two-stage self-control model proposed by Myrseth and Fishbach (2009), the process of self-control is divided into two stages. Stage 1 involves identifying conflicting contradictions related to self-control, while Stage 2 entails using self-control strategies to resolve these conflicts. Specifically, when an individual faces temptation, they must first identify whether giving in to indulgence conflicts with their long-term goals (Stage 1). If it is determined that a conflict exists, the individual will employ a series of self-regulation strategies to resist the immediate temptation, ensuring the fulfillment of their long-term goals (Stage 2). In other words, individuals who experience high levels of depletion and are in anonymous situations tend to lower their self-control standards. The inherent hidden nature of anonymity allows them to engage in indulgent behaviors, such as deception, without conflicting with their long-term goal of becoming a good person. We thus hypothesize that:

*H2: Anonymity exacerbates the after-effects of ego depletion and increases deceptive behavior.*

### 1.4 Ego depletion and deceptive behaviors: moral emotions

The reason why individuals with high self-depletion are more prone to engage in deceptive behaviors in anonymous situations, compared to non-anonymous ones, is that their level of control diminishes in such contexts. For them acts of deception and other immoral behaviors do not conflict with their image of being a moral person.So, how can we reduce immoral behavior in such situations? This brings us to our second factor: moral emotions. Moral emotions are social emotions that arise when individuals evaluate their own or others’ thoughts and behaviors based on certain social norms and moral standards (Rudolph and Tscharaktschiew, 2014). The process model of ego depletion proposed by Inzlicht et al. (2014) suggests that ego depletion does not result from a limited self-control resource but rather from shifts in attention and motivation following self-control exertion. Specifically, after exerting self-control, individuals’ task motivation shifts from obligatory “have-to” goals to more intrinsically rewarding “want-to” goals. For example, Wagner et al. (2013) found that when food-related stimuli were presented to dieters in a state of ego depletion, the brain regions activated were associated with reward processing. This suggests that prior self-control tasks heightened sensitivity to reward stimuli, shifted motivation, and reduced self-control, ultimately increasing the likelihood of dishonest behavior. Consequently, if moral emotions are activated to shift motivation back from “want-to” to “have-to” the effects of ego depletion may be alleviated to some extent, thereby reducing deceptive behavior.

Moral emotions are also composite emotions and can be classified as either positive or negative depending on their valence (Eisenberg, 2000). When an individual’s external behavior aligns with or violates their internal moral standards, it triggers corresponding positive or negative moral emotions. As is widely known, an individual’s cognition can, to some extent, determine the generation of emotions. However, have you ever considered that emotions can also influence cognition? Fredrickson and Joiner (2002) found that individuals who maintain positive emotions during difficult times are more likely to view the situation from different perspectives and consider various angles. This phenomenon may be due to the fact that positive emotions expand an individual’s momentary thought-action repertoire, prompting a broader range of thinking and behavior than usual, thus enhancing their self-control resources. Positive moral emotions motivate individuals to engage in actions that benefit others and society, while negative moral emotions cause individuals to stop harmful behaviors and engage in moral compensation actions (Tangney et al., 2007). In other words, both positive and negative moral emotions promote prosocial behavior and reduce the incidence of deceptive behavior (Hoffman, 2000). Based on the above, we propose Hypothesis that:

*H3: Both positive and negative moral emotions can alleviate the after-effects of ego depletion and reduce deceptive behavior.*

## 2 Method

### 2.1 Participants

In this study, we use G * Power 3. 1. 9. 2 to calculate the required sample size (Faul et al., 2007). Based on previous research on ego-depletion (Brown et al., 2020; Giboin and Wolff, 2019; Mangin et al., 2021; McMorris et al., 2018), the present study assumes the effect size *f* = 0.40. Experiment 1 employs a two-factor completely randomized design with the following parameters: effect size *f* = 0.40, *α* = 0.05, power (1 - *β*) = 0.95, number of groups = 4, numerator degrees of freedom (*df*) = 3 (number of groups - 1). The required sample size for this experiment is 112 participants. Experiment 2 also follows a two-factor completely randomized design, with effect size *f* = 0.40, *α* = 0.05, power (1 - *β*) = 0.95, number of groups = 6, numerator degrees of freedom (*df*) = 5 (number of groups - 1). The required sample size for this experiment is 130 participants.

#### 2.1.1 Experiment 1

The influence of anonymity on deceptive behaviors under different ego depletion situations. A total of 120 non-psychology majors (from freshman to graduate second grade) were recruited in an elective course at a university in Heilongjiang Province, with an average age of (20 ± 2) years old, including 63 males and 57 females. The experimenter manually assigned participants to one of four groups using random allocation: high-depletion anonymous group, high-depletion non-anonymous group, low-depletion anonymous group, and low-depletion non-anonymous group, with 30 participants in each group. There were no statistically significant differences in age, *F* (3, 116) = 0.09, *p* = 0.963, *η_p_^2^ =* 0.001, or gender, *F* (3, 116) = 0.03, *p* = 0.992, *η_p_^2^ =* 0.001, distribution among the groups.

#### 2.1.2 Experiment 2

The influence of moral emotion on deceptive behavior under different ego depletion situations. A total of 150 non-psychology majors (from freshman to graduate second grade), 72 males and 78 females, with an average age of (20 ± 2) years old, were selected from a university in Heilongjiang Province by means of WeChat group and posters. The experimenter manually assigned participants to one of six groups using random allocation: high-depletion positive moral emotion group, high-depletion negative moral emotion group, high-depletion neutral emotion group, low-depletion positive moral emotion group, low-depletion negative moral emotion group, and low-depletion neutral emotion group, with 25 participants in each group. There were no statistically significant differences in age, *F*(5, 144) = 0.45, *p* = 0.814, *η_p_^2^ =* 0.02, or gender, *F*(5, 144) = 0.06, *p* = 0.997, *η_p_^2^ =* 0.01, distribution among the groups.

None of the participants had a history of neurological or psychiatric disorders, normal or corrected visual acuity, no color blindness, and had not participated in similar experiments before. Informed consent was signed before the experiment, and certain remuneration was obtained after the experiment.

### 2.2 Procedure

#### 2.2.1 Experiment 1

First, the participant entered the laboratory and were asked to complete the modified “Breaking the Habit Paradigm” - cross out “e” task (Boucher and Kofos, 2012). In the first phase, participants in both the high-depletion group and the low-depletion group were instructed to cross out every instance of the letter “e.” The task instructions for both groups were as follows: “This task is designed to assess your attention to detail. Please mark all occurrences of the letter ‘e’ in the passage using ‘/’ without missing any. Complete the task as quickly and accurately as possible.” In the second phase, the low-depletion group continued with the same task as in the first phase, receiving the instruction: “Please continue marking the letter ‘e’ as quickly and accurately as possible.” However, participants in the high-depletion group were required to override the previously established response pattern, inhibiting their automatic tendency to mark every “e” in order to induce ego depletion. Their revised instructions were: “Please continue marking the letter ‘e’ but refrain from marking it under the following two conditions: (1) If the letter ‘e’ is immediately followed by a vowel (a, e, i, o, u); (2) If a vowel appears in the second letter before ‘e’ (i. e., there is one intervening letter between ‘e’ and the vowel).” This modification increased cognitive load and self-regulatory effort in the high-depletion group, as they had to override the prepotent response established in the first phase, leading to a greater degree of ego depletion compared to the low-depletion group. After completing the task, participants responded to retrospective questions regarding ego depletion (Englert and Bertrams, 2016). They were asked to rate the following on a 7-point Likert-type scale: (1) “After completing this task, how fatigued did you feel?” (1 = “Not at all fatigued,” 7 = “Extremely fatigued”); (2) “How much effort did you exert to complete this task?” (1 = “No effort at all,” 7 = “Maximum effort”); and (3) “To what extent did you feel your energy was depleted after performing this task?” (1 = “Not depleted at all,” 7 = “Extremely depleted”).

Second, different anonymous groups were guided into the corresponding laboratories. Each participant in the anonymous group experimented in a separate laboratory (That is, the visual-perception task is performed in a separate laboratory, providing data on deception behavior), and they were explicitly told that none of the other participants knew his/her real identity and behaviors; all participants in the non-anonymous group experimented in the same laboratory (That is, the visual-perception task is performed in the same laboratory that provides the data on deception behaviors), and they were explicitly told that his/her identity and behaviors were known by the other participants and they were required to enter their real names at the beginning of the visual-perceptual task, and whose names appeared in the upper left corner of the screen throughout the task.

Third, the participants were guided through a visual-perception task. A modified visual-perception task (Chance et al., 2011) was used in this study to examine deceptive behaviors (number of deceptive behaviors, deception tendencies). In this task, the participants were shown a square picture divided by a diagonal line into the left and right sides, with 30 red dots unevenly distributed on both sides. In the experiment, a total of 200 trials were divided into 2 groups, each group had 100 trials, in which 25 trials had more red dots on the left; 25 had more red dots on the right; and the other 50 had the same number of red dots on the left and right sides. The participants’ task was to determine which side contained more red dots. When a participant believed that the left side had more red dots, they pressed the “1″ key; when they thought the right side had more red dots, they pressed the “2″ key. Each press of the “1″ key yielded a fixed reward of 0.5 yuan, and each press of the “2″ key yielded a fixed reward of 0.01 yuan. The final compensation for each participant was calculated by multiplying the number of times they pressed each key by the fixed reward associated with that key, and then adding the resulting amounts together. In each group, if there were 25 trials with more red dots on the left, then the participant pressed the “1″ button, which indicates that the participant is honest; if there were 25 trials with more red dots on the right, and the participant still pressed the “1″ button, indicating that the participant is deceiving at this time, which is recorded as the time of deceptive behaviors; in another 50 trials with the equal number of red dots on the right and left side, if the participant pressed the “1″ button more often, it indicated that the participant tended to do deceptive behaviors, The number of deception tendencies is the number of times the “1” key is pressed. In addition, E-prime also recorded the choice reaction time of participants in 4 groups to complete the visual-perception task, that is the time it takes for participants with different ego-depletion to perform the deceptive behaviors. After completing the visual-perception task, participants were asked a retrospective question regarding anonymity (Wang, 2018): “To what extent do you think the other participants in the experiment were able to discern your identity and behavior?” (1 = “not at all,” 7 = “completely”). Finally, the participants filled out the Moral Identity Measure.

#### 2.2.2 Experiment 2

First, the participants entered the laboratory and were asked to complete the modified Stroop task (Gailliot et al., 2007). This task employed as the loss task of self-control, four Chinese characters (red, green, yellow and blue) were written in four colors respectively, which were divided into two kinds of stimuli: color consistent with word meaning and color inconsistent with word meaning. In the formal experiment, a total of 120 stimuli were presented in both the high-depletion group and the low depletion group, the high-depletion group presented 48 stimuli with consistent color-word and 72 stimuli with inconsistent color-word. In the low depletion group, 72 stimuli with consistent color-word were presented, 48 stimuli with inconsistent color-word were presented. Exposing participants in the low-depletion group to incongruent color-word stimuli helps prevent the introduction of extraneous variables such as boredom, which could otherwise confound the experimental results. In this study, the Stroop task was written with E-Prime2.0, and the experimental process was as follows: instruction - fixation point “+” (200 ms) - target stimulus (1,000 ms) - empty screen (500 ms-1,000 ms), and the background of all experimental stimuli was black. Experimental requirements are as follows: Press F button when the target stimulus word color is the same; Press J button when the word color is inconsistent, no press or miss press is treated as an error response. After completing the task, participants were asked to answer retrospective questions regarding ego depletion (Englert and Bertrams, 2016). They were asked to rate the following on a 7-point Likert-type scale: (1) “After completing this task, to what extent did you feel fatigued?” (1 = “Not at all fatigued,” 7 = “Extremely fatigued”); (2) “How much effort did you invest in suppressing the influence of word meaning on color naming?” (1 = “No effort at all,” 7 = “All of my effort”); and (3) “After completing this task, to what extent did you feel that your energy was depleted?” (1 = “Not at all depleted,” 7 = “Extremely depleted”).

Second, participants in each group were guided through a tailored induction procedure designed to elicit the specific moral emotions corresponding to their assigned condition. In Experiment 2, a modified “behavioral recall paradigm” (Sachdeva et al., 2009) was employed to induce moral emotions. Three distinct emotional conditions were used, each associated with five specific words. For positive moral emotions, the words were: caring, generosity, fairness, friendliness, and dedication; for negative moral emotions, the words were: betrayal, greed, meanness, selfishness, and deception; and for neutral emotions, the words were: books, keys, houses, chairs, and furniture. Participants in each group were first instructed to copy the set of words corresponding to their assigned moral emotion four times while reflecting on each word’s meaning. After completing this copying task, they were asked to write a personal narrative about a past event in which they used each of these words at least once, describing both the event’s details and the emotions they experienced at that time. In addition, they completed the Positive Affect and Negative Affect Scale (PANAS).

Third, participants were guided to complete the visual-perceptual task (identical to that used in Experiment 1). Finally, they completed the Moral Identity Measure (MIM).

### 2.3 Measures

#### 2.3.1 Moral identity measure (MIM)

In experiments 1 and 2, the Moral Identity Measure (Wen, 2012) was used to test the level of moral identity, that is, the degree of self-identification of moral qualities, including 5 moral qualities of honesty, trustworthiness, responsibility, sincerity and integrity, divided into 5 dimensions of emotional identification (e.g., “These qualities make me happy”), cognitive identification (e.g., “It’s important to me to be a person who possesses these qualities”), attitude identification (e.g., “I have a strong desire to possess these qualities”), behavioral identification (e.g., “The things I do clearly reflect that I have these qualities”) and external identification (e.g., “I’ve been accused by others of having these qualities”), with a total of 20 items. 5-point Likert-type scale were used to measure (1 = “completely disagree,” 5 = “Fully agree”) the level of moral identity. The Cronbach’s *α* for Experiments 1 and 2 were 0.85 and 0.82, respectively.

Moral self-identity refers to the extent to which individuals recognize and accept their own moral image as part of their personal identity. This construct reflects a long-term process of moral internalization, making it a relatively stable trait that does not change rapidly over time. Nevertheless, research has shown that moral self-identity influences both prosocial behavior (Aquino and Reed, 2002) and deceptive behavior (Gino et al., 2011). Consequently, this study controls for moral self-identity as a extraneous variable.

#### 2.3.2 Positive affect and negative affect scale (PANAS)

In Experiment 2, we employ the scale that was modelled on the PANAS by Watson et al. (1988) and localised in China (Qiu et al., 2008) and based on previous research (Ren et al., 2014) to measure the emotional state of the participants, including glad, happy, excited, delighted, joyful 5 positive emotions (e.g., “The level of glad you are feeling now is”) and sad, angry, afraid, nervous, grieved 5 negative emotions (e.g., “The level of sad you are feeling now is”). We also use 5-point Likert-type scale to evaluate (1 = “weak,” 5 = “strong”) the degree of positive or negative emotions. In Experiment 2, the Cronbach’s *α* for positive affect was 0.92, and the Cronbach’s *α* for negative affect was 0.89.

## 3 Results

### 3.1 Experiment 1: the effect of anonymity on deceptive behavior under different self-depletion contexts

#### 3.1.1 Manipulate checks

The high depletion group average scored significantly higher on the retrospective ego depletion question compared to the low depletion group [(5.6 ± 0.6) vs. (2.7 ± 0.9)], *t*(118) = 22.09, *p* < 0.001, Cohen’s *d* = 0.89, indicating that the “cross-out ‘e’” task effectively induced ego depletion to some extent.

The anonymity manipulation check revealed that the anonymity recall scores were significantly lower in the anonymous group than in the non-anonymous group [(2.6 ± 0.9) vs. (5.9 ± 0.9)], *t*(118) = 19.19, *p* < 0.001, Cohen’s *d* = 0.87, suggesting that the manipulation of anonymity was successful to some extent.

There were no statistically significant differences in moral identity scores across the 4 groups (see Table 1), indicating that the participants’ moral attitudes were similar and ruling out the effect of the level of trait integrity of the participants on the results of the experiment.

**Table 1:** Test of difference in moral attitudes (M ± SD).

Moral identity	High depletion anonymous group	High depletion non-anonymous group	Low depletion anonymous group	Low depletion non-anonymous group	*F*	*p*	*η_p_^2^*	95%*CI*
	*n* = 30	*n* = 30	*n* = 30	*n* = 30				
Emotional identity	4.3 ± 0.3	4.2 ± 0.3	4.2 ± 0.3	4.2 ± 0.4	0.80	0.499	0.02	[0.00, 0.07]
Cognitive identity	4.3 ± 0.4	4.2 ± 0.5	4.3 ± 0.4	4.2 ± 0.5	0.39	0.759	0.01	[0.00, 0.05]
Attitudinal identity	4.1 ± 0.6	3.9 ± 0.6	4.0 ± 0.6	3.9 ± 0.5	0.61	0.612	0.02	[0.00, 0.06]
Behavioral identity	4.3 ± 0.3	4.2 ± 0.4	4.3 ± 0.4	4.2 ± 0.4	0.92	0.433	0.02	[0.00, 0.08]
External recognition	4.0 ± 0.5	3.8 ± 0.6	4.0 ± 0.5	3.7 ± 0.6	1.72	0.166	0.04	[0.00, 0.11]

#### 3.1.2 ANOVA for deceptive behaviors

Experiment 1 employed a 2 (ego depletion: high vs. low) × 2 (anonymity: anonymous vs. non-anonymous) between-participants design. The dependent variables were the experimental measures of deceptive behavior, including the number of deceptions, the tendency to deceive, and the choice response time (in ms). The study examined the effect of anonymity on college students’ deceptive behavior under varying levels of ego depletion by comparing these measures across the groups. Table 2 presents the data for the number of deceptions, the tendency to deceive, and the corresponding choice response times for the four groups.

**Table 2:** The number of deceptive behaviors by the 4 participant groups (M ± SD).

Group	Number of deceptions	Choice reaction time/ms	Deceptive tendencies	Choice reaction time/ms
High depletion anonymous group (*n* = 30)	39.1 ± 9.6	341.0 ± 22.3	71.1 ± 13.3	333.9 ± 32.6
High depletion non-anonymous group (*n* = 30)	29.5 ± 5.4		55.9 ± 7.5	
Low depletion anonymous group (*n* = 30)	14.8 ± 4.1	362.2 ± 18.3	43.2 ± 7.5	353.3 ± 28.6
Low depletion non-anonymous groups (*n* = 30)	12.3 ± 4.8		38.4 ± 10.5	

An analysis of variance (ANOVA) was conducted on the results of the four participant groups. The main effect of ego depletion was statistically significant for both the number of deceptions, *F*(1, 116) = 320.77, *p* < 0.001, *η_p_^2^* = 0.73, and the tendency to deceive, *F*(1, 116) = 154.30, *p* < 0.001, *η_p_^2^* = 0.57. Participants in the high-depletion group engaged in significantly more deceptive behaviors and exhibited a stronger tendency to deceive than those in the low-depletion group.

Furthermore, significant differences were observed in response times for both the number of deceptions, *t*(118) = 5.68, *p* < 0.001, Cohen’s *d* = 0.46, and the tendency to deceive, *t*(118) = 3.46, *p* = 0.001, Cohen’s *d* = 0.30, between participants with different levels of ego depletion. Specifically, the high-depletion group demonstrated shorter response times when making deceptive choices compared to the low-depletion group.

A significant main effect of anonymity was found for both the number of deceptions, *F*(1, 116) = 27.38, *p* < 0.001, *η_p_^2^* = 0.19, and the tendency to deceive, *F*(1, 116) = 29.74, *p* < 0.001, *η_p_^2^* = 0.20. Participants in the anonymous condition exhibited a higher frequency of deception and a greater tendency to deceive than those in the non-anonymous condition.

Additionally, a significant interaction effect was found between ego depletion and anonymity for both the number of deceptions, *F*(1, 116) = 9.64, *p* = 0.002, *η_p_^2^* = 0.08, and the tendency to deceive, *F*(1, 116) = 8.10, *p* = 0.005, *η_p_^2^* = 0.07. When ego depletion was high, participants in the anonymous condition engaged in significantly more deceptive behaviors, *F*(1, 116) = 34.75, *p* < 0.001, *η_p_^2^* = 0.23, and exhibited a greater tendency to deceive, *F*(1, 116) = 34.44, *p* < 0.001, *η_p_^2^* = 0.23, compared to those in the non-anonymous condition. However, when ego depletion was low, there were no significant differences between the anonymous and non-anonymous groups in terms of the number of deceptions, *F*(1, 116) = 2.26, *p* = 0.135, *η_p_^2^* = 0.02, or the tendency to deceive, *F*(1, 116) = 3.40, *p* = 0.068, *η_p_^2^* = 0.03.

### 3.2 Experiment 2: the effect of moral emotions on deceptive behaviors under different Ego depletion situations

#### 3.2.1 Manipulate checks

The high depletion group average scored higher on the ego depletion retrospective question than the low depletion group [(5.4 ± 0.7) vs. (3.1 ± 0.7), *t*(148) = 20.87, *p* < 0.001, Cohen’s *d* = 0.86], suggesting that the Stroop task successfully triggered ego depletion to some extent.

Based on previous research (Fei et al., 2016), the effectiveness of moral emotion induction was assessed by comparing mood differences among participants in each group after the induction of different moral emotions. The differences in moral emotions were statistically significant for both positive and negative emotions (see Table 3). Further *post hoc* analyses using the least significant difference (LSD) test revealed that the differences in both positive and negative emotions for each of the three moral emotions were statistically significant, suggesting that the moral emotion induction was effective to some extent.

**Table 3:** Comparative table of positive and negative emotions for different moral emotions.

Emotional type	Types of moral emotions	*M ± SD*	*F*	*p*	*η_p_^2^*	95%*CI*
Positive emotions	Positive moral emotions	4.2 ± 0.4	627.38	0.000	0.89	[0.86, 0.91]
	Negative moral emotions	1.9 ± 0.3				
	Neutral emotions	3.4 ± 0.3				
Negative emotions	Positive moral emotions	2.2 ± 0.6	271.93	0.000	0.79	[0.73, 0.83]
	Negative moral emotions	4.1 ± 0.3				
	Neutral emotions	2.8 ± 0.3				

No significant differences in moral identity scores were found across the 6 groups of participants (see Table 4), suggesting that there were no significant differences in the self-identification of moral qualities, thus ruling out the potential influence of trait integrity on the experimental results.

**Table 4:** Test of difference in moral attitudes (M ± SD).

Moral identity	High-depletion positive moral emotions group	Low-depletion positive moral emotions group	High-depletion negative moral emotions group	Low-depletion negative moral emotions group	High-depletion neutral emotions group	Low-depletion neutral emotions group	*F*	*p*	*η_p_^2^*	95%CI
	*n* = 25	*n* = 25	*n* = 25	*n* = 25	*n* = 25	*n* = 25				
Emotional identity	3.9 ± 0.3	3.8 ± 0.3	3.9 ± 0.3	4.0 ± 0.4	4.0 ± 0.2	4.0 ± 0.3	1.64	0.153	0.05	[0.00, 0.11]
Cognitive identity	4.0 ± 0.4	4.0 ± 0.4	4.0 ± 0.4	3.9 ± 0.3	3.9 ± 0.4	4.0 ± 0.4	0.50	0.778	0.02	[0.00, 0.04]
Attitudinal identity	3.8 ± 0.5	3.8 ± 0.4	3.7 ± 0.6	3.7 ± 0.6	3.7 ± 0.5	3.8 ± 0.6	0.21	0.959	0.01	[0.00, 0.01]
Behavioral identity	4.1 ± 0.4	4.0 ± 0.4	4.2 ± 0.3	4.1 ± 0.3	4.1 ± 0.3	4.2 ± 0.3	1.57	0.174	0.05	[0.00, 0.11]
External recognition	3.8 ± 0.5	3.8 ± 0.4	3.9 ± 0.4	3.9 ± 0.3	3.8 ± 0.4	3.9 ± 0.4	0.40	0.849	0.01	[0.00, 0.03]

#### 3.2.2 ANOVA for deceptive behaviors

Experiment 2 employed a 2 (ego depletion: high vs. low) × 3 (moral emotion: positive vs. Negative vs. neutral) between-participants design. The dependent variables were deceptive behaviors, including the number of deceptions and the tendency to deceive. The effect of moral emotion on deceptive behavior under different ego depletion conditions was examined by comparing the differences in deceptive behaviors across the various groups. The results for the number of deceptions and deception tendency across the 6 groups are summarized in Table 5.

**Table 5:** Number of deceptive behaviors in the 6 participant groups (M ± SD).

Group	Number of deceptions	Deceptive tendencies
High-depletion positive moral emotions group (*n* = 25)	26.1 ± 7.1	54.6 ± 9.3
High-depletion negative moral emotions group (*n* = 25)	33.0 ± 6.7	60.8 ± 9.8
High-depletion neutral emotions group (*n* = 25)	39.5 ± 5.6	69.2 ± 7.8
Low-depletion positive moral emotions group (*n* = 25)	5.4 ± 2.6	41.1 ± 9.3
Low-depletion negative moral emotions group (*n* = 25)	9.6 ± 3.9	45.5 ± 6.2
Low-depletion neutral emotions group (*n* = 25)	7.6 ± 2.7	44.2 ± 4.4

ANOVA was conducted to examine the effects of ego depletion and moral emotions on the number of deceptions and the tendency to deceive across the six participant groups. The results indicated that the main effect of ego depletion was statistically significant for both the number of deceptions, *F*(1, 144) = 930.07, *p* < 0.001, *η_p_^2^* = 0.87, and the tendency to deceive, *F*(1, 144) = 185.27, *p* < 0.001, *η_p_^2^* = 0.56. Participants in the high-depletion group engaged in significantly more deceptive behaviors and exhibited a stronger tendency to deceive compared to those in the low-depletion group.

The main effect of moral emotions was also statistically significant for both the number of deceptions, *F*(2, 144) = 30.78, *p* < 0.001, *η_p_^2^* = 0.30, and the tendency to deceive, *F*(2, 144) = 15.51, *p* < 0.001, *η_p_^2^* = 0.18. *Post hoc* analyses using the LSD test revealed that all three moral emotion groups significantly differed from one another in both the number of deceptions and the tendency to deceive. Specifically, participants in the positive moral emotion group exhibited the lowest levels of deception, followed by those in the negative moral emotion group, with the neutral emotion group displaying the highest levels of deception.

Additionally, a significant interaction effect was found between ego depletion and moral emotions for both the number of deceptions, *F*(2, 144) = 16.78, *p* < 0.001, *η_p_^2^* = 0.19, and the tendency to deceive, *F*(2, 144) = 7.42, *p* < 0.001, *η_p_^2^* = 0.09.

To further examine this interaction, a simple effects analysis was conducted. Results indicated that in the high-ego depletion condition, pairwise comparisons revealed that all three moral emotion groups significantly differed from one another in both the number of deceptions, *F*(2, 144) = 43.38, *p* < 0.001, *η_p_^2^* = 0.38, and the tendency to deceive, *F*(2, 144) = 20.92, *p* < 0.001, *η_p_^2^* = 0.23. Specifically, participants in the positive moral emotion group exhibited the lowest levels of deceptive behavior, followed by those in the negative moral emotion group, with the neutral emotion group displaying the highest levels of deception.

In the low-ego depletion condition, only the difference in the number of deceptions between the positive moral emotion group and the negative moral emotion group was statistically significant, *t*(48) = 4.45, *p* < 0.001, Cohen’s *d* = 1.26, indicating that participants with positive moral emotions engaged in significantly fewer deceptive behaviors than those with negative moral emotions. However, there were no statistically significant differences in the number of deceptions between the neutral emotion group and either the positive moral emotion group (*p* = 0.428) or the negative moral emotion group (*p* = 0.475). Although these results were not statistically significant, the mean trend suggested that participants in the negative moral emotion group engaged in slightly more deceptive behaviors than those in the neutral emotion group, whereas participants in the positive moral emotion group engaged in slightly fewer deceptive behaviors than those in the neutral emotion group.

No statistically significant differences were found among the moral emotion groups in terms of the tendency to deceive, *F*(2, 144) = 2.01, *p* = 0.137, *η_p_^2^* = 0.03. However, the mean trend suggested a pattern in which participants in the positive moral emotion group exhibited the lowest tendency to deceive, followed by those in the neutral emotion group, with the negative moral emotion group showing the highest tendency.

## 4 Discussion

In this study, we have investigated the effects of anonymity and moral emotions on college students’ deceptive behaviors under different ego depletion situations. Our findings show that the results of Experiments 1 and 2 both partially verified hypothesis 1, and the number of deception and deception tendencies of the high depletion group were more than the low depletion group, which was consistent with the results of previous research (Stucke and Baumeister, 2010). In Experiment 1, the participants in the high depletion group had lower decision-making responses than those in the low depletion group, which partially verified hypothesis 1 and was in line with the theory of the dual system model of ego depletion. After the participants had undergone ego depletion, the remaining self-control resources were not enough to support their moral judgement, rational decision, and other cold system behaviors, and then in the hot system, which did not take up the control resources for automated responses, instinctive impulses and egoism are aroused to a higher degree, so the participants with high ego depletion are prone to deceptive behaviors. Self-control and deception were further considered to investigate the underlying mechanisms. Effective self-control contains three main components: standards, monitoring and power. Standards are the internal requirements of external social norms and moral standards that individuals are expected to act according to Balliet and Joireman (2010); monitoring is the continuous tracking of the parts of the behaviors that need to be changed to meet the requirements (Carver and Scheier, 1981); power is similar to the resources of psychological control, which plays a role in changing the individual’s behaviors and the current state, as well as in the comprehension and processing of information. Therefore, when the individual is in a state of ego depletion after-effects, firstly, the participant is unable to rationally understand the information and judge whether the current situation is in line with their ethical norms and requirements; secondly, although the participant still has the appropriate standards of their behaviors, he/she does not have enough power to adjust and change his/her behaviors, and at the same time cannot inhibit the self-interested tendencies, and it is more likely to happen the deception of behavior and other immoral behaviors.

In Experiment 1, the deceptive behavior in the anonymous group was higher than in the non-anonymous group. In high-depletion contexts, the deceptive behavior in the anonymous group was greater than in the non-anonymous group, while in low-depletion contexts, there was no statistically significant difference in deceptive behavior between the anonymous and non-anonymous groups. This finding supports Hypothesis 2, showing that anonymity may have exacerbated the aftereffects of ego depletion, potentially leading to an increase in participants’ deceptive behavior. According to the theory of energy depletion (Baumeister et al., 2000), an individual’s psychological energy is finite, and repeated use within a given time can lead to energy depletion, resulting in a self-depletion aftereffect. When an individual is in an anonymous situation and experiences high self-depletion, their limited self-control resources lead them to make the most cost-effective choice, which is to deceive; When individuals experience a low level of ego depletion, their self-control resources remain sufficient to support a series of moral judgments driven by the “cold” cognitive system. In other words, this is because in an anonymous context, the individual’s deceptive behavior does not affect how others perceive them; in other words, their current choice does not conflict with their long-term goals. In other words, anonymity influences an individual’s behavior by affecting their self-control level.

When an individual is in a situation where deceptive behaviors and other immoral actions are likely to occur, yet we require the individual to act honestly, introducing another factor—moral emotions—can effectively resolve this conflict. In Experiment 2, we found that deception behaviors were significantly lower in the positive moral emotion group than in the negative moral emotion group, which, in turn, exhibited significantly lower levels of deception than the neutral emotion group. In the high ego depletion situations, the deceptive behaviors of the positive moral emotions group were smaller than the negative moral emotions group and smaller than the neutral emotions group; in the low ego depletion situations, there was no difference in the deceptive behaviors of the three groups of participants, which verified hypothesis 3, that the moral emotions may have mitigated the aftereffects of ego depletion, potentially leading to a reduction in participants’ deceptive behavior, which is consistent with the previous study (Fei et al., 2016). This result also provides experimental evidence for the resource-allocation model of self-control (Beedie and Lane, 2012), which proposed that an individual’s psychological energy is not depleted and can be induced by external means. Inducing positive moral emotions to reduce an individual’s unethical behaviors can also be explained from the following perspectives: firstly, it is believed that positive emotions alleviate the ego depletion after-effects by replenishing an individual’s depleted psychological control resources, and secondly, Fredrickson and Branigan (2005) proposed in the emotion broaden-and-build theory that positive emotions facilitate an individual’s access to a range of resources, which enhances his or her physiological resilience to recover depleted psychological energy in a shorter period. Inducing negative moral emotions to reduce an individual’s unethical behavior can be explained by the moral compensation theory (Zhong et al., 2010a, 2010b), where negative moral emotions create pressure on the individual to adjust to the moral imbalance, and the individual then reduces the pressure by reducing deceptive behaviors or increasing altruistic behaviors and other moral compensatory behaviors in subsequent behaviors.

In summary, through 2 experiments, this study provides evidence that high ego depletion contexts may increase individuals’ deceptive behavior, anonymity may exacerbate the aftereffects of ego depletion, potentially leading to an increase in deceptive behavior, and moral emotions may alleviate the aftereffects of ego depletion, potentially leading to a reduction in deceptive behavior. This is very important to reduce the occurrence of deceptive behavior in real life and to ensure the peace and stability of society. In a series of scenarios requiring honesty and trustworthiness such as family and school education, interpersonal interactions, and judicial hearings, try to keep the individual in a lower state of ego depletion, and if the individual is already in a high state of ego depletion, one is to ensure that the external environment is not anonymous and reduce the awakening of the individual’s selfishness and egoistic instincts, thus decreasing the probability of deceptive behavior, and the second is to induce moral emotions to the individual, stimulate his remaining psychological energy, self-control the individual and reduce the occurrence of deceptive behaviors.

There are some limitations in this research. Trait self-control and other inherent individual characteristics can influence state ego depletion (Dvorak and Simons, 2009). Future research on state ego depletion should consider incorporating trait self-control and other dispositional factors as extraneous variable. In evaluating the effectiveness of our moral emotion manipulation, relying solely on the measurement of positive and negative affect (e.g., via the PANAS) as a substitute for directly assessing the manipulation of positive and negative moral emotions is clearly insufficient—this has also been a persistent issue in prior research on moral emotions. In future studies, we propose supplementing the PANAS with an additional assessment paradigm. After completing the behavioral recall paradigm, participants will be asked, using a 5-point Likert-type scale, to rate the extent to which the story they wrote reflects how they view themselves as (1) a student, (2) a member of an organization, (3) a moral person, and (4) safety conscious. The scores on item 3 will then be compared across the positive moral emotion, negative moral emotion, and neutral emotion conditions. In combination with the PANAS results, this will allow for a more comprehensive evaluation of the effectiveness of the moral emotion manipulation.

The sample in the present study primarily consisted of college students. However, as individuals grow older, they experience changes in self-control that may affect the results of this study. Therefore, future research should examine the relationship between ego depletion and deceptive behavior across different age groups to enhance the external validity of our findings. Additionally, pre-registration before conducting experiments is crucial for empirical research, as it helps reduce publication bias, enhance reproducibility, and improve research transparency. Future empirical studies should adopt pre-registration practices to strengthen the reliability and credibility of findings.

## Data Availability

The raw data supporting the conclusions of this article will be made available by the authors, without undue reservation.
